# Cuevaenes C–E: Three new triene carboxylic derivatives from *Streptomyces* sp. LZ35Δ*gdmAI*

**DOI:** 10.3762/bjoc.10.82

**Published:** 2014-04-15

**Authors:** Jing-Jing Deng, Chun-Hua Lu, Yao-Yao Li, Shan-Ren Li, Yue-Mao Shen

**Affiliations:** 1 Key Laboratory of Chemical Biology (Ministry of Education), School of Pharmaceutical Sciences, Shandong University, No. 44 West Wenhua Road, Jinan, Shandong 250012, P. R. China

**Keywords:** antibacterial activity, cuevaenes, geometrical isomer, natural products, *Streptomyces* sp. LZ35

## Abstract

Two pairs of geometrical isomers – cuevaenes A (**1**) and C (**3**) as well as cuevaenes D (**4**) and E (**5**) – and cuevaene B (**2**) were isolated from *gdmAI*-disrupted *Streptomyces* sp. LZ35. The constitution of cuevaene C (**3**) was found to be identical to cuevaene A (**1**) by means of NMR spectroscopy and high resolution mass spectrometry. However, the relative configurations of the triene side chain moieties were determined to be different. It was established on the basis of spectroscopic data that cuevaenes D (**4**) and E (**5**) are amides and geometrical isomers. Cuevaenes A–C (**1–3**) displayed moderate activity against Gram-positive bacteria (e.g., *Bacillus subtilis* strain ATCC 11060) and modest activity against fungi (e.g., *Fusarium verticillioides* strain S68 and *Rhizoctonia solani* strain GXE4). However, cuevaenes D (**4**) and E (**5**) showed no inhibitory activity against any of the tested microbes.

## Introduction

Cuevaenes are polyketides containing 3-hydroxybenzoic acid (3-HBA). Only 4 naturally occurring cuevaenes are described. Cuevaenes A and B were isolated from *Streptomyces* sp. HKI 0180 fifteen years ago and display moderate antibacterial activity against Gram-positive bacteria [1]. JBIR-23 and JBIR-24, novel anti-malignant pleural mesothelioma (MPM) agents, were isolated from *Streptomyces* sp. AK-AB27 six years ago [2]. All of these natural products have a tricyclic core and a polyene side chain with an enolmethyl ether inside. This type of triene structural moiety is rarely found in natural products.

**Figure 1:**
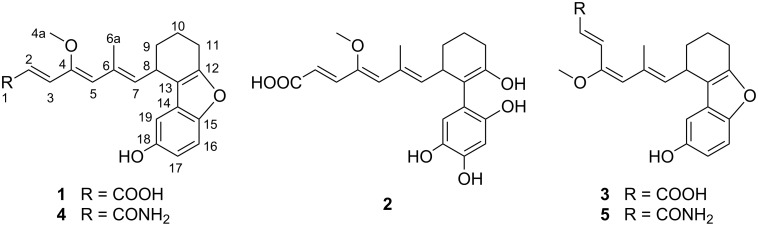
Cuevaenes A–E (**1–5**) isolated from *Streptomyces* sp. LZ35.

*Streptomyces* sp. LZ35 was isolated from the intertidal soil collected at Jimei, Xiamen, China [3]. An orphan type I polyketide synthase gene cluster that contains a putative chorismatase/3-hydroxybenzoate synthase gene was identified by genome sequencing and bioinformatics analysis of *Streptomyces* sp. LZ35. This finding suggests the potential of LZ35 to produce 3-HBA-containing polyketides [4]. A *gdmAI* (encoding the first geldanamycin PKS module) deletion strain LZ35Δ*gdmAI* was constructed [3,5], as the production of geldanamycins was much higher than other constituents in this strain. This mutant could provide a relatively “clean” background to facilitate the isolation of minor constituents. Here, we present the isolation and structural characterization of three new 3-HBA-containing polyketides, namely cuevaenes C–E (**3–5**), along with cuevaenes A and B (**1** and **2**) from the metabolites of the strain LZ35Δ*gdmAI* (Figure 1). The antimicrobial activities of cuevaenes A–E (**1**–**5**) were evaluated.

## Results and Discussion

The strain *Streptomyces* sp. LZ35Δ*gdmAI* was cultured on oatmeal medium for 11 days at 28 °C and extracted with EtOAc/MeOH/AcOH (80:15:5, v/v/v) to afford a dark crude extract that was partitioned between petroleum ether and MeOH. The MeOH extract was subjected to column chromatography over Sephadex LH-20 and RP-18 silica gel. The pure compounds of cuevaenes A–E were finally obtained by reversed-phase HPLC (Supporting Information File 1, Figure S1).

Compound **1** was isolated as a needle crystal. The HRMS–ESI spectra shows a quasi-molecular ion [M + H] ^+^ at *m*/*z* 355.1544 consistent with the molecular formula C_21_H_22_O_5_ (calcd for C_21_H_22_O_5_, 355.1540). Compound **2** was isolated as a light yellow powder. The HRMS–ESI spectra shows a quasi-molecular ion [M + H] ^+^ at *m*/*z* 389.4107 consistent with the molecular formula C_21_H_24_O_7_ (calcd for C_21_H_24_O_7_, 389.4111). The structures of **1** and **2** were elucidated on the basis of spectroscopic data. The comparison of these spectroscopic data to those in the literature readily revealed that compounds **1** and **2** are identical to cuevaenes A and B, the triene carboxylic acids originally isolated from *Streptomyces* sp. HKI0180 [1,6–7].

**Figure 2:**
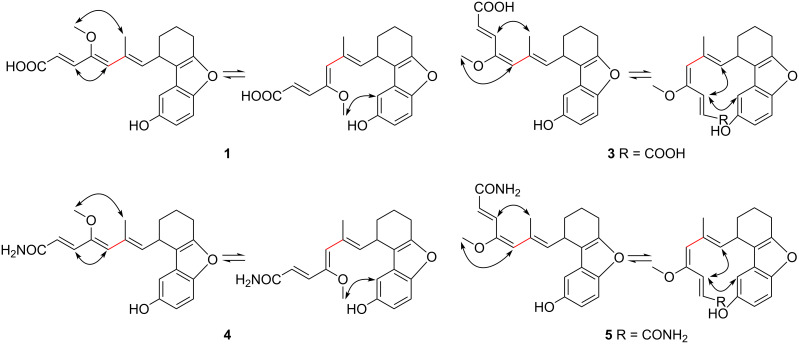
Selected NOESY correlations of compounds **1**, **3** and **4**, **5**.

Compound **3** was obtained as a light yellow powder. The HRMS–ESI spectra shows a quasi-molecular ion [M + H] ^+^ at *m*/*z* 355.1544. This is consistent with the molecular formula C_21_H_22_O_5_ (calcd for C_21_H_22_O_5_, 355.1540) and identical to the measurement of cuevaene A (**1**). The direct connectivity between protons and carbons were established by HSQC experiments (Table 1). Further NMR studies included ^1^H,^1^H COSY and HMBC and established the constitution of **3**, which is identical to the constitution of **1**. The relative configuration of **3** was determined by the proton coupling constants and NOE correlations observed in NOESY experiments. The geometrical configuration of the triene side chain moiety in **3** was established to be 2*E*, 4*E*, and 6*E* by the proton spin coupling constant between 2-H and 3-H (*J* = 15.4 Hz) and the strong NOE correlations from 3-H to 6a-H_3_, 7-H and19-H, and from 4a-H_3_ to 5-H. Two conformations were observed in the NOE spectrum of **3** due to the rotation around the C-5/C-6 single bond (Figure 2). Therefore, compound **3** was determined to be the geometrical isomer of **1**.

**Table 1:** ^1^H and ^13^C NMR spectroscopy data for compounds **3**, **4** and **5**^a^.

Pos.	**3** (MeOD)		**4** (CDCl_3_)		**5** (MeOD)	
		
	δ_H_ (mult., *J* Hz)	δ_C_	δ_H_ (mult., *J* Hz)	δ_C_	δ_H_ (mult., *J* Hz)	δ_C_

1	–	170.9 (s)	–	168.7 (s)	–	171.4 (s)
2	6.12 (d, 15.4)	119.6 (d)	6.05 (d, 15.2)	118.0 (d)	6.37 (d, 15.2)	121.4 (d)
3	7.64 (d, 15.4)	138.6 (d)	6.96 (d, 15.2)	140.5 (d)	7.60 (d, 15.2)	136.0 (d)
4 4a	– 3.66 (s, 3H)	152.8 (s) 55.4 (q)	– 3.58 (s)	151.7 (s) 60.3 (q)	– 3.66 (s, 3H)	152.8 (s) 55.3 (q)
5	5.71 (s)	115.5 (d)	5.60 (s)	129.9 (d)	5.68 (s)	114.8 (d)
6 6a	– 2.05 (s, 3H)	132.1 (s) 18.1 (q)	– 2.15 (s)	131.8 (s) 15.1 (q)	– 2.03 (s, 3H)	132.5 (s) 18.1 (q)
7	5.29 (d, 9.9)	136.4 (d)	5.53 (d, 9.9)	139.4 (d)	5.32 (d, 9.6)	135.6 (d)
8	3.82 (m)	34.0 (d)	3.76 (m)	32.5 (d)	3.81 (m)	34.1 (d)
9	2.08 (m)	31.6 (t)	1.92 (m)	29.8 (t)	2.09 (m)	31.6 (t)
	1.68 (m)		1.43 (m)		1.64 (m)	
10	2.08 (m)	22.5 (t)	2.01 (m)	21.7 (t)	2.11 (m)	22.7 (t)
	1.89 (m)		1.81 (m)		1.89 (m)	
11	2.69 (m, 2H)	24.3 (t)	2.69 (m, 2H)	23.5 (t)	2.70 (m, 2H)	24.3 (t)
12	–	155.9 (s)	–	155.2 (s)	–	156.0 (s)
13	–	115.9 (s)	–	114.7 (s)	–	116.1 (s)
14	–	130.3 (s)	–	129.1 (s)	–	130.2 (s)
15	–	153.7 (s)	–	151.8 (s)	–	153.8 (s)
16	7.15 (d, 8.7)	111.7 (d)	7.21 (d, 7.6)	111.3 (d)	7.16 (d, 8.7)	111.8 (d)
17	6.63 (br d)	112.2 (d)	6.70 (dd, 2.2, 7.6)	111.7 (d)	6.63 (dd, 2.5, 8.7)	112.3 (d)
18	–	150.3 (s)	–	149.3 (s)	–	150.3 (s)
19	6.75 (br s)	105.2 (d)	6.74 (br s)	104.7 (d)	6.84 (d, 2.5)	105.3 (d)

^a^Data were obtained at 600 MHz for ^1^H and 151 MHz for ^13^C NMR.

Compounds **4** and **5** showed positive color reactions with acid-modified Dragendorff's reagent on TLC. Their molecular formulas were determined to be C_21_H_23_NO_4_ by HRMS–ESI (*m*/*z* 354.4193 [M + H] ^+^, calcd 354.4190). The IR spectra of **4** and **5** showed absorptions for a primary amide at 3347 and 3220 cm^−1^. The C=O group of an amide absorbs at 1660 cm^−1^, conjugated C=C groups at 1615 cm^−1^, and aromatic ring C=C groups at 1592 and 1461 cm^−1^. The ^13^C NMR spectra recorded for **4** and **5** shows 21 resonances (1 × CH_3_; 1 × OCH_3_, 3 × CH_2_; 8 × CH; 8 × C), which are similar to those of **1** (Table 1). Consequently, the constitution of **4** and **5** were determined to be the amides of **1** and **3**, respectively. The geometrical configuration of the triene side chain of **4** was established to be 2*E*, 4*Z*, and 6*E* by the proton spin coupling constant between 2-H and 3-H (*J* = 15.2 Hz) and the strong NOE correlations from 3-H to 5-H and from 4a-H_3_ to 6a-H_3_ and 19-H (Figure 2). The triene side chain of **5** was established to be 2*E*, 4*E*, and 6*E* by the proton spin coupling constant between 2-H and 3-H (*J* = 15.2 Hz) and the strong NOE correlations from 3-H to 6a-H_3_, 7-H and 19-H and from 4a-H_3_ to 5-H. Two conformations were observed for both compounds **4** and **5** due to the rotation around the C-5/C-6 single bond (Figure 2).

The antimicrobial activities of cuevaenes A–E (**1–5**) were investigated by paper disc diffusion assay. Cuevaenes A–C (**1–3**) displayed moderate activity against Gram-positive bacteria such as *Bacillus subtilis* strain ATCC 11060 (diameters of inhibitory zones 11.5, 12.3 and 14.2 mm, respectively) at 30 μg/disc, and modest activity against fungi such as *Fusarium verticillioides* strain S68 and *Rhizoctonia solani* strain GXE4 at 30 μg/disc. Interestingly, cuevaenes D (**4**) and E (**5**) showed no inhibitory activity against all tested microbes at 30 μg/disc, indicating that the existence of the carboxyl group (1-COOH) is essential for the bioactivity.

Previously, Liu et al. [7] reported the synthesis of cuevaene A. However, the NMR spectroscopic data of cuevaene A failed to match the published data. In a later paper, Taylor et al. [6] reported the total synthesis of cuevaene A by another synthetic route, which determined the true structure of cuevaene A (**1**). They also explained that Liu et al. failed to match the published data due to their usage of a different solvent. Besides, the Shin-ya group also revised the structure of cuevaene A as compound **1** [8]. Recently, we have reported the identification and characterization of the biosynthetic gene cluster of cuevaenes from *Streptomyces* sp. LZ35. Cuevaenes are 3-HBA-containing type I polyketides. An aromatic starter unit (3-HBA converted from chorismate) and six extender units are employed in the polyketide chain extension. In addition, several post-PKS modifications are required to complete the biosynthesis of cuevaenes [4]. In this study, two pairs of geometrical isomers were reported. The difference between the geometrical isomers is the stereoconfiguration of the Δ4,5 double bond. A previous study indicated that the double-bond geometry is determined by the stereochemistry of Ketoreductase (KR)-catalyzed ketoreduction [9]. By multiple sequence alignment of modular PKS KR domains we identified an Asp residue in the KR5 domain of cuevaenes PKS (Supporting Information File 1, Figure S22). This Asp residue occurs in all ketoreduction catalyzing KRs that are known to produce the D configuration). This finding suggests the formation of a *trans* double bond catalyzed by dehydratase (DH) [10]. As assumed, the isolated compounds **4** and **5** exhibited a *trans* Δ4,5 configuration. However, the main products **1**, **2** and **3** we obtained contain the *cis* Δ4,5 configuration, which need to be further explored.

## Experimental

**General experimental procedures**. NMR spectra were measured on Bruker DRX-600 MHz NMR spectrometer (Bruker Daltonics Inc., Billerica, Massachusetts) with tetramethylsilane (TMS) as an internal standard. HRMS–ESI were carried out on an LTQ-Orbitrap XL. Optical rotations were measured on a GYROMAT-HP polarimeter. The IR spectra (KBr) were obtained on a Nicolet iN10 Micro FTIR Microscope (Thermo Scientific). Reversed-phase (RP) C18 silica gel for column chromatography was obtained from Merck (Darmstadt, Germany) and Sephadex LH-20 from GE Amersham Biosciences (Piscataway, New Jersey). Silica gel (200–300 mesh) for column chromatography and silica gel GF_254_ for TLC were purchased from Qingdao Marine Chemical Ltd. (Qingdao, China). Semipreparative HPLC were performed on an Agilent 1260 equipped with a ZORBAX XDB-C18 5 μm column (9.4 × 250 mm). All solvents used were of analytical grade. Compounds were visualized under UV light and by spraying with H_2_SO_4_/EtOH (1:9, v/v) followed by heating and acid-modified Dragendorff's reagent (5 mL of 40% (w/v) potassium iodide solution was added to 5 mL of 1.7% (w/v) bismuth nitrate solution in 20% (v/v) acetic acid, and the mixture was diluted to 100 mL with 10% (w/v) sulfuric acid).

**Material.** Strain LZ35 was isolated from the intertidal soil collected at Jimei, Xiamen, China. It was identified as a *Streptomyces* species according to the 16S rRNA sequence (accession number JX853780). The mutant strain LZ35Δ*GdmAI* (SR101) was constructed by deleting the first PKS module of the geldanamycin biosynthesis gene [5].

**Cultivation, extraction and isolation**: The *GdmAI* deletion mutant strain SR101 was cultured in petri dishes laid with ca. 20 mL oatmeal medium (oatmeal 30 g, saline salt 1 mL, agar 20 g, pH 7.2) with a total volume of 30 litres for 11 days at 28 °C. The culture was diced and extracted three times overnight with AcOEt/MeOH/AcOH 80:15:5 (v/v/v) at room temperature and patitioned between H_2_O and EtOAc until the EtOAc layer was colorless. Then, the EtOAc extract was dried with Na_2_SO_4_, and the solvent was removed under vacuum. The EtOAc extract was partitioned with petroleum ether and MeOH until the petroleum ether layer was colorless. The MeOH extract (5.0 g) was subjected to Sephadex LH-20 eluted with MeOH to obtain 6 fractions, i.e., Fr. 1–6. Fr. 5 (400 mg) was further subjected to MPLC over RP-18 silica gel 80 g. 76 subfractions with 16 mL for each gradient were obtained from the elution with 50% MeOH (1–18 tube), 60% MeOH (19–30 tube), 70% MeOH (31–52 tube), 80% MeOH (53–64 tube), and 100% MeOH (65–76 tube) in water, respectively. According to TLC results, 1–32, 33–52, 53–59, 60–63 and 64–76 were combined and marked as Fr. 5a, Fr. 5b (25 mg), Fr. 5c (30 mg), Fr. 5d (8.0 mg) and Fr. 5e, respectively. Fr. 5b (25 mg) was purified by semipreparative reversed-phase HPLC (ZORBAX Eclipse XDB-C18 5 μm,column ID: 9.4 × 250 mm, flow rate: 4 mL/min, elution: MeOH/H_2_O (45–55, v/v), UV detections at 274 nm) to afford **4** (*t*_R_ 4.7 min, 7 mg), **3** (*t*_R_ 6.8 min, 4 mg) and **5** (*t*_R_ 7.6 min, 1 mg). Fr. 5c (30 mg) was purified by semipreparative reversed-phase HPLC (ZORBAX Eclipse XDB-C18 5 μm, column ID: 9.4 × 250 mm, flow rate: 4 mL/min, elution: CH_3_CN /H_2_O (62–38, v/v), UV detections at 274 nm) to yield **1** (*t*_R_ 7.2min, 18 mg). Fr. 5d (8 mg) was finally purified by semipreparative reversed-phase HPLC (ZORBAX Eclipse XDB-C18 5 μm, column ID: 9.4 × 250 mm, flow rate: 4 mL/min, elution: CH_3_CN /H_2_O (70–30, v/v), UV detections at 274 nm) to yield **2** (*t*_R_ 7.6 min, 2 mg).

**Cuevaene C (3)**: light yellow powder; [a]_D_^20^ +230.0 (*c* 0.175, MeOH); HRMS–ESI *m/z*: [M + H]^+^ calcd for C_21_H_22_O_5_, 355.1540; found, 355.1544; IR (KBr) *v*_max_: 3278, 1686, 1619, 1596, and 1463 cm^−1^. See Table 1 for the NMR data.

**Cuevaene D (4)**: light yellow powder; [a]_D_^20^ +130.0 (*c* 0.315, MeOH); HRMS–ESI *m/z*: [M + H]^+^, (calcd for C_21_H_23_NO_4_, 354.4190; found, 354.4193; IR (KBr) *v*_max_: 3347, 3220, 1660, 1615, 1591, and 1461 cm^−1^. For the NMR data see Table 1.

**Cuevaene E (5)**: white oil; [a]_D_^20^ +25.0 (*c* 0.08, MeOH); HRMS–ESI *m/z*: [M + H]^+^, (calcd for C_21_H_23_NO_4_, 354.4190; found, 354.4193; IR (KBr) *v*_max_: 3338, 3220, 1660, 1592, and 1461 cm^−1^. For the NMR data see Table 1.

**Antibacterial and antifungal bioassays**: Antibacterial and antifungal activities of compounds **1–5** were tested by the paper disc diffusion method [11]. The bacterial *Bacillus subtilis* strain ATCC 11060 was grown on LB agar. The fungus *Fusarium verticillioides* strain S68 and *Rhizoctonia solani* strain GXE4 were grown on potato dextrose agar. Test compounds were absorbed onto individual paper disks (6 mm diameter) at 30 μg/disc and placed on the surface of the agar. The assay plates were incubated at 28 °C for 48 h for antifungal activity and at 37 °C for 24 h for antibacterial activity and examined for the presence of a zone of inhibition.

## Supporting Information

File 1 Spectroscopic data and other relevant information for cuevaenes A–E (**1–5**).
