# Supplementary material for: Cuevaenes C–E: Three new triene carboxylic derivatives from Streptomyces sp. LZ35ΔgdmAI
Source: Beilstein J Org Chem. 2014 Apr 15;10:858–62. doi: 10.3762/bjoc.10.82 (PMC3999817; doi:10.3762/bjoc.10.82)
Supplement: File 1 — Spectroscopic data and other relevant information for cuevaenes A–E (1–5). [file Beilstein_J_Org_Chem-10-858-s001.pdf]

Supporting information

for

Cuevaenes C–E: Three new triene carboxylic derivatives from *Streptomyces* sp. LZ35Δ*gdmAI*

Jing-Jing Deng[§], Chun-Hua Lu[§], Yao-Yao Li, Shan-Ren Li and Yue-Mao Shen*

Address: Key Laboratory of Chemical Biology (Ministry of Education), School of Pharmaceutical Sciences, Shandong University, No. 44 West Wenhua Road, Jinan, Shandong 250012, P. R. China

Email: Yue-mao Shen* - yshen@sdu.edu.cn

* Corresponding Author

[§]These two authors contributed equally to this work.

Spectroscopic data and other relevant information

for cuevaenes A–E (1–5)

Figure S1 HPLC data of compounds **1–5**……………………………………………………S3

Figure S2 ^{1}H NMR (600 MHz, CD_3OD) spectrum for compound **1**…………………………..S4

Figure S3 ^{13}C NMR (151 MHz, CD3OD) spectrum for compound **1**………………………...S4

Figure S4 The HSQC spectrum for compound **1**………………………………………………S5

Figure S5 The HMBC spectrum for compound **1**………………………………………………S5

Figure S6 ^{1}H/^{1}H COSY spectrum for compound **1**……………………………...……………S6

Figure S7 The NOE spectrum for compound **1**………………………………………………..S6

Figure S8 ^{1}H NMR (600 MHz, CD_3OD) spectrum for compound **2**…………………………..S7

Figure S9 The NOE spectrum for compound **2**………………………………………………..S7

Figure S10 ^{1}H NMR (600 MHz, CD_3OD) spectrum for compound **3**………..………………S8

Figure S11 ^{13}C NMR (151 MHz, CD3OD) spectrum for compound **3**……....………………S8

Figure S12 The HSQC spectrum for compound **3**……………………………………………S9

Figure S13 The HMBC spectrum for compound **3**……………………………………………S9

Figure S14 ^{1}H/^{1}H COSY spectrum for compound **3**……………………………….………...S10

Figure S15 The NOE spectrum for compound **3**……………………………………………..S10

Figure S16 ^{1}H NMR (600 MHz, $CDCl_3$) spectrum for compound **4**………....……………S11

Figure S17 ^{13}C NMR (151 MHz, $CDCl_3$) spectrum for compound **4**……....……………....S11

Figure S18 The NOE spectrum for compound **4**…………………………….……………S12

Figure S19 ^{1}H NMR (600 MHz, CD_3OD) spectrum for compound **5**………………………..S12

Figure S20 ^{13}C NMR (151 MHz, CD3OD) spectrum for compound **5**……...…………….S13

Figure S21 The NOE spectrum for compound **5**……………………………………………..S13

Figure S22 Alignment of modular PKS Ketoreductase (KR) domains……………………..S14

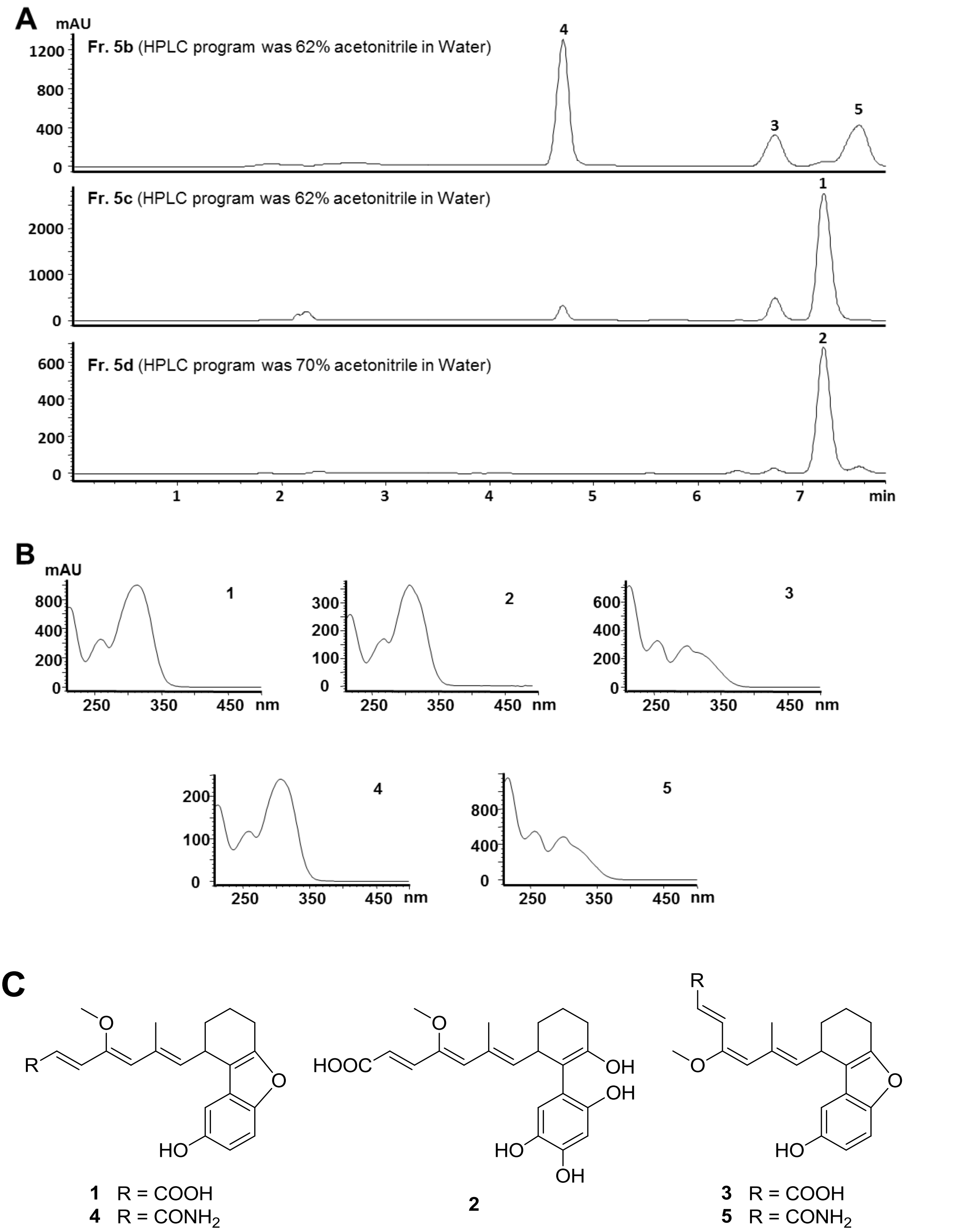

Figure S1: **A**: HPLC analysis of the constituents of Fr. 5b, Fr.5c and Fr.5d (HPLC were performed on an Agilent 1260 equipped with a YMC-C18 5 μm column (4.6 × 250 mm) and detected at 274 nm on a UV detector. **B**: Ultraviolet absorption curve of compounds **1–5**. **C**: Chemical structure of compounds **1–5**.

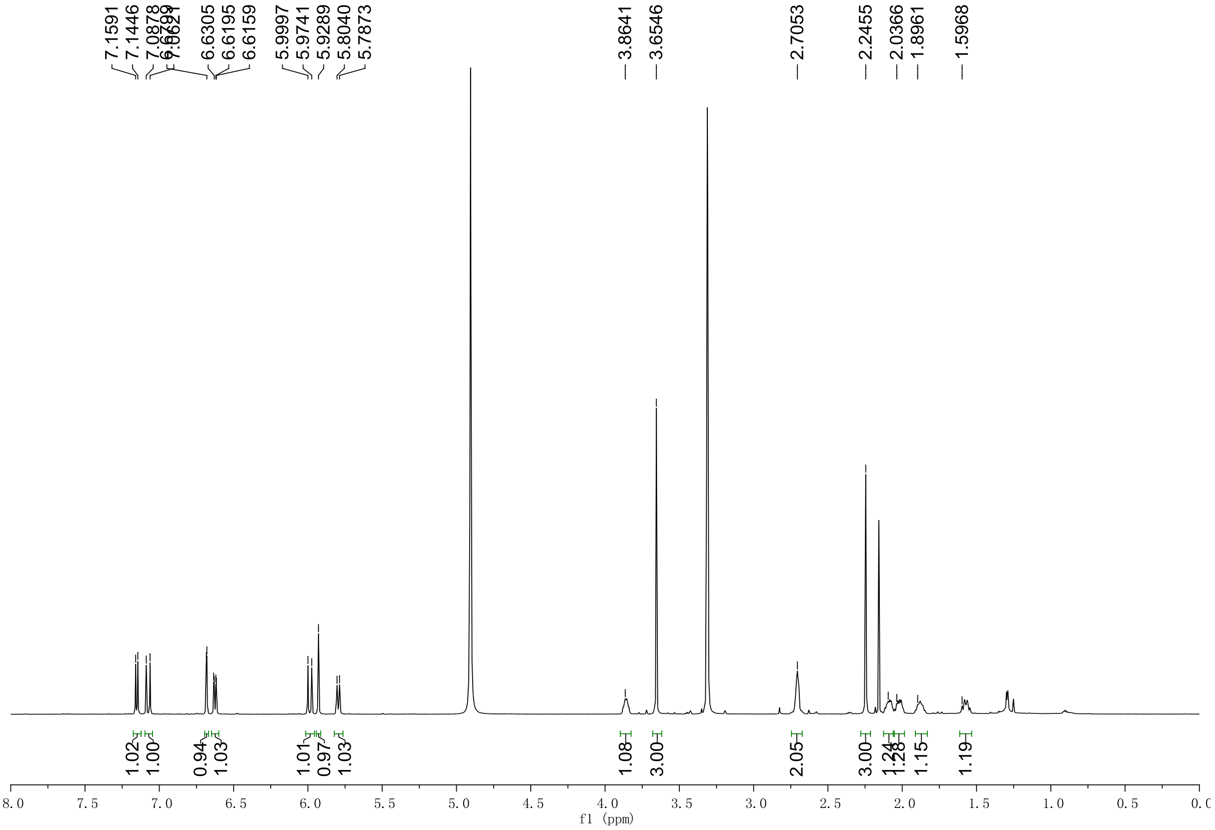

Figure S2: ^{1}H NMR (600 MHz, CD_3OD) spectrum for compound **1**.

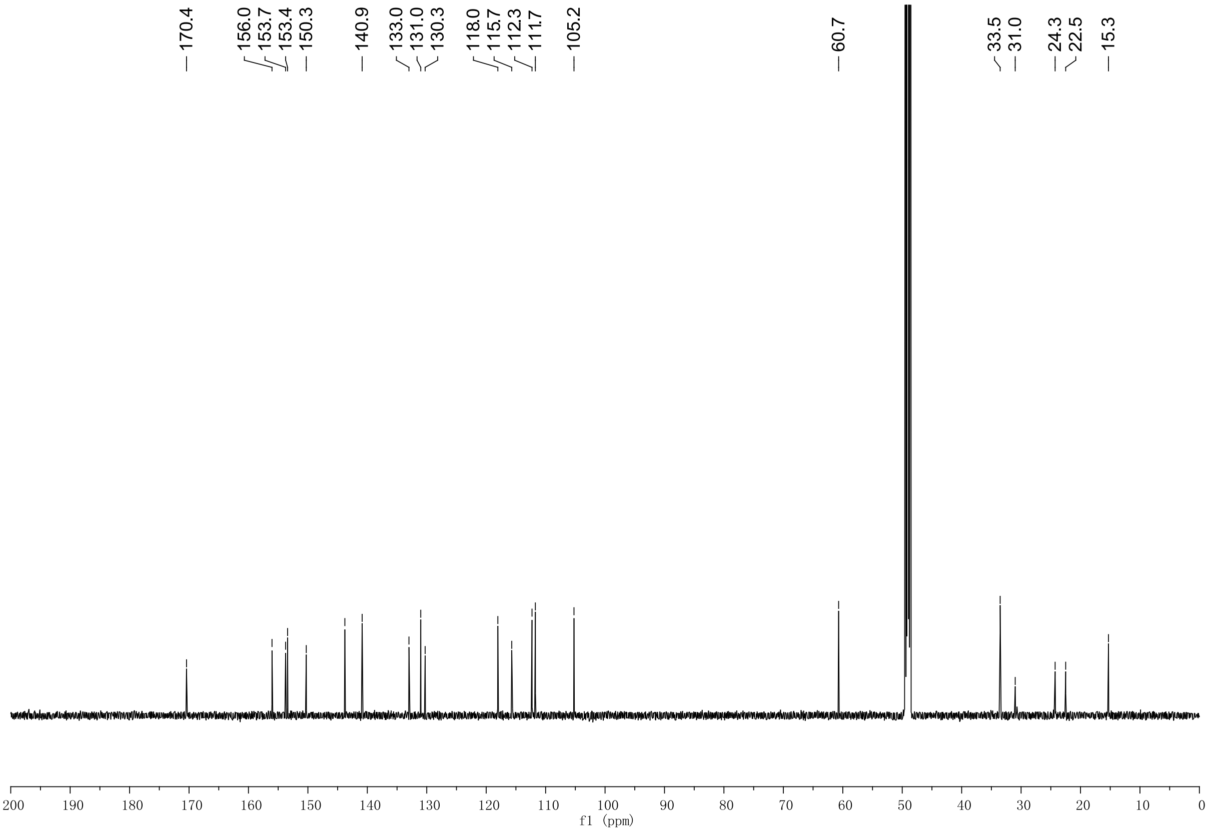

Figure S3: ^{13}C NMR (151 MHz, CD3OD) spectrum for compound **1**.

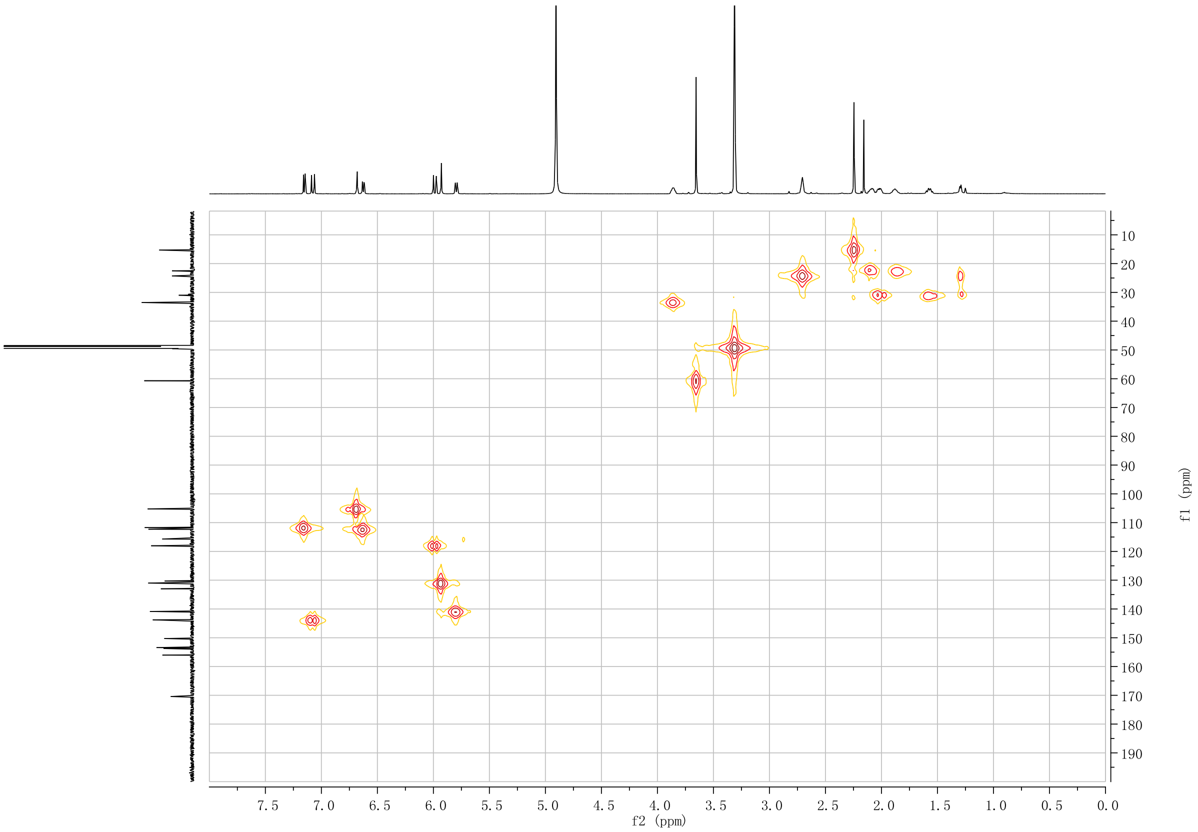

Figure S4: The HSQC spectrum for compound **1**.

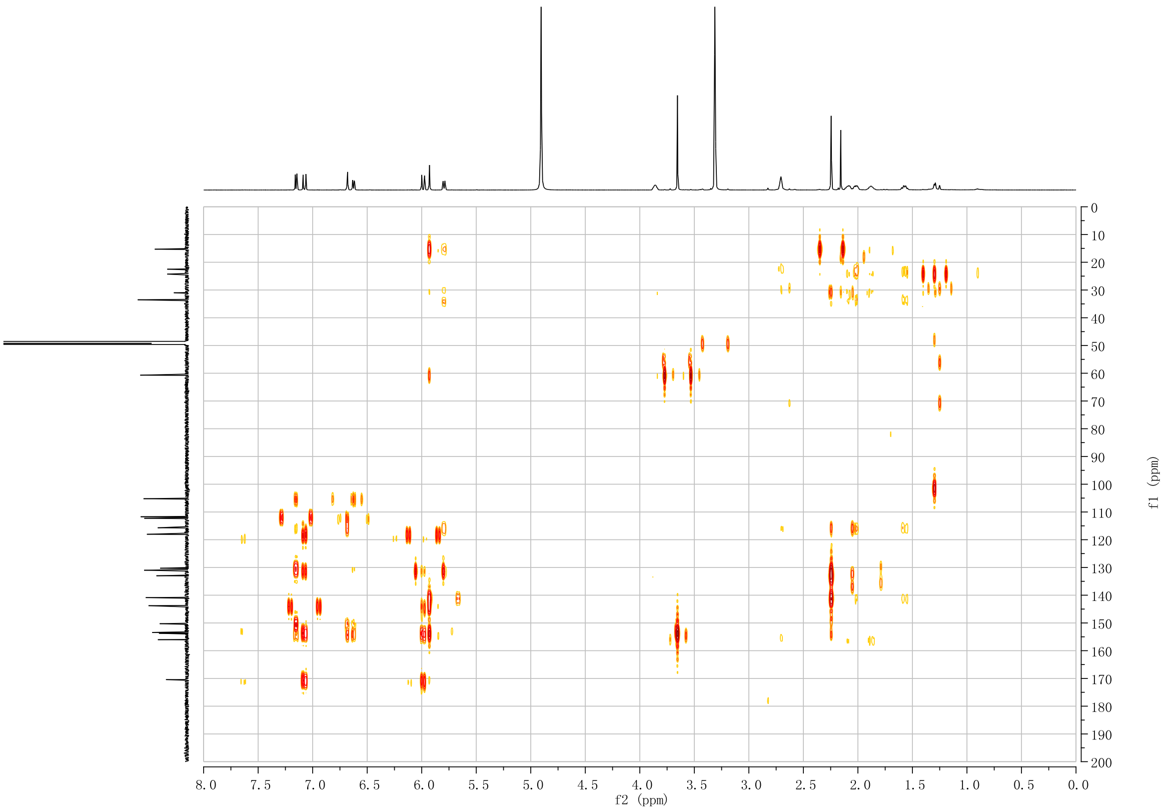

Figure S5: The HMBC spectrum for compound **1**.

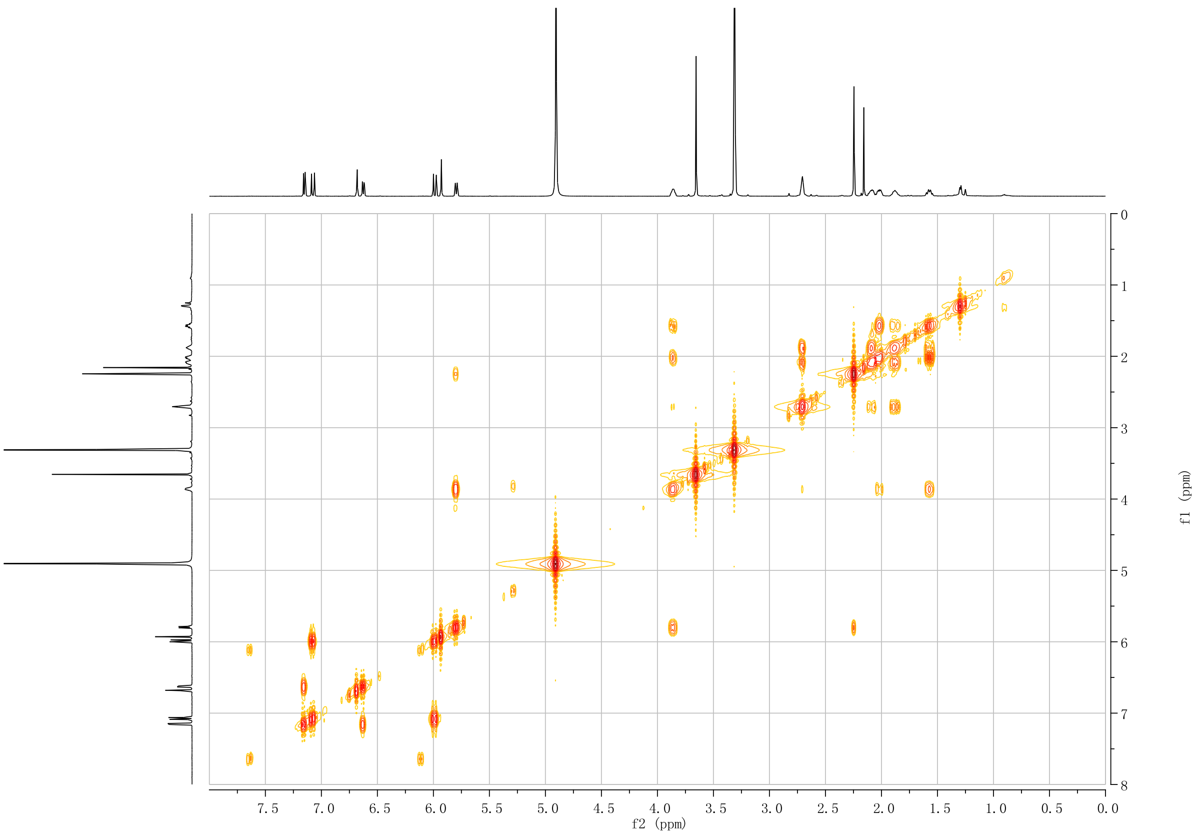

Figure S6: $^1H/^1H$ COSY spectrum for compound **1**.

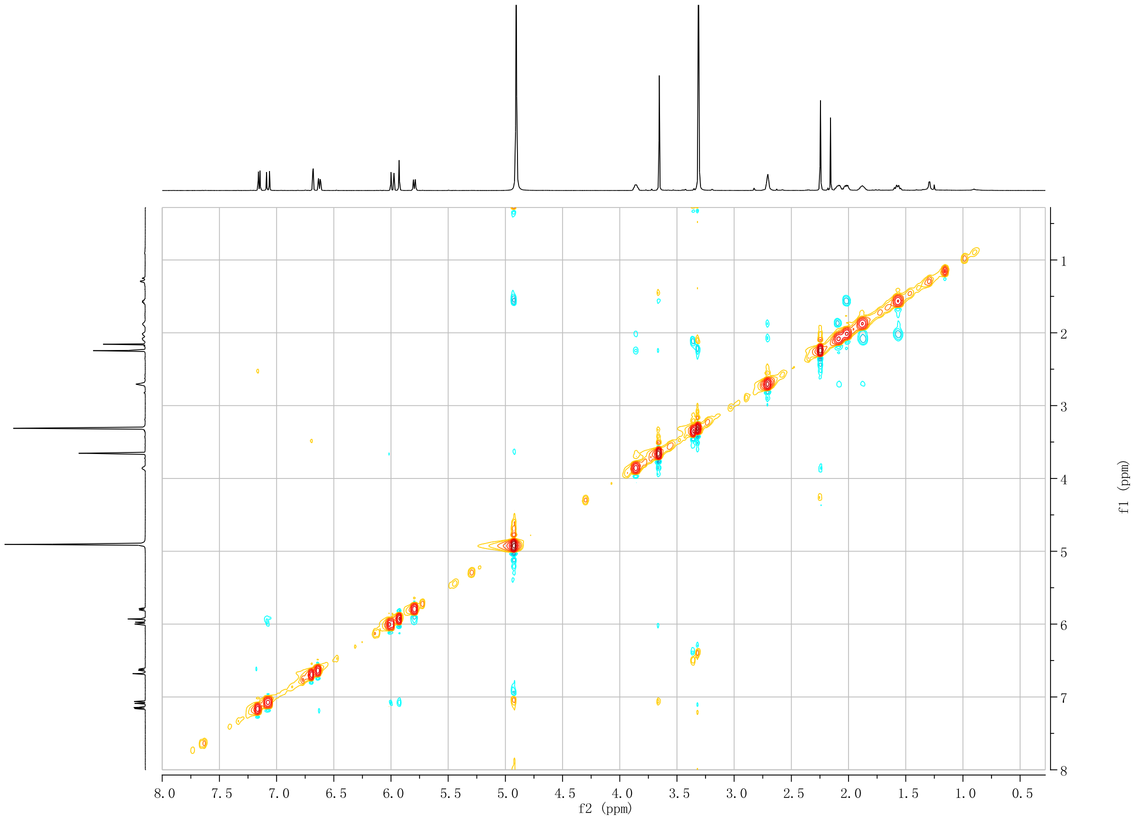

Figure S7: The NOE spectrum for compound **1**.

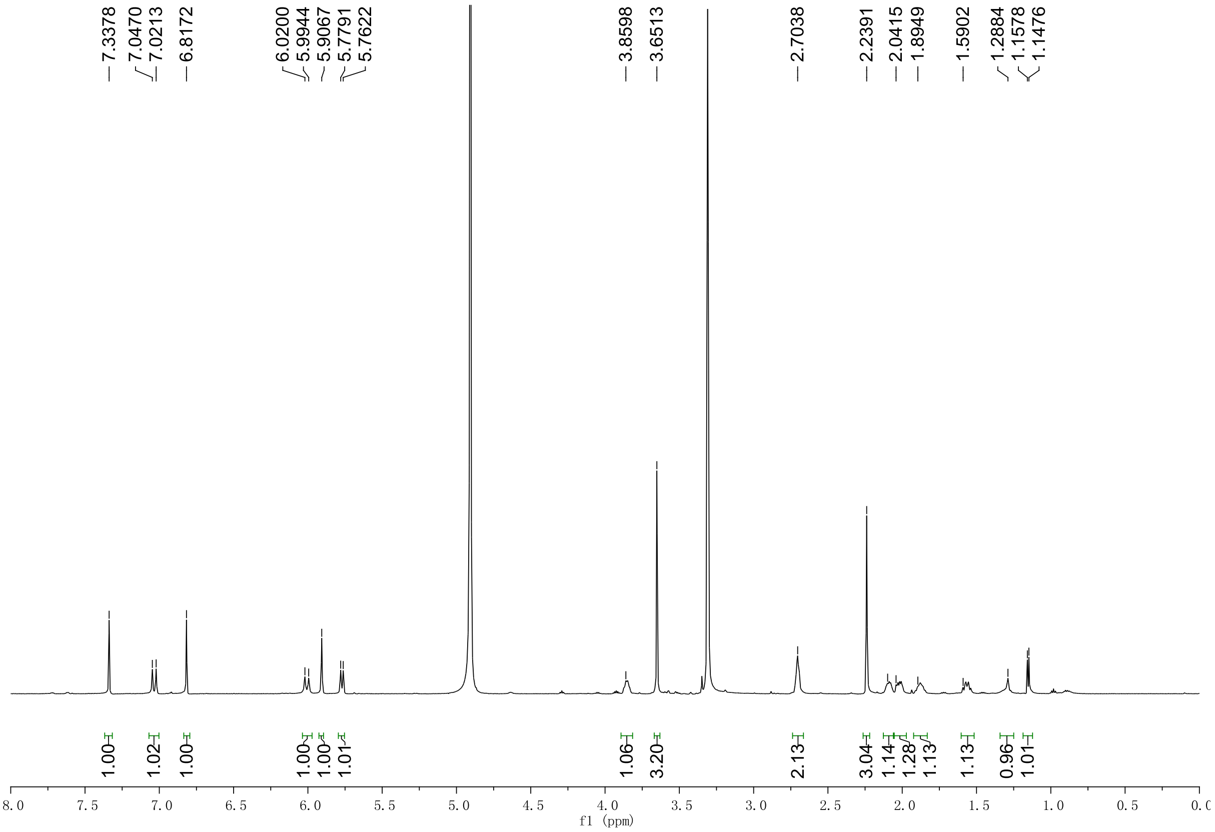

Figure S8: ^{1}H NMR (600 MHz, CD_3OD) spectrum for compound **2**.

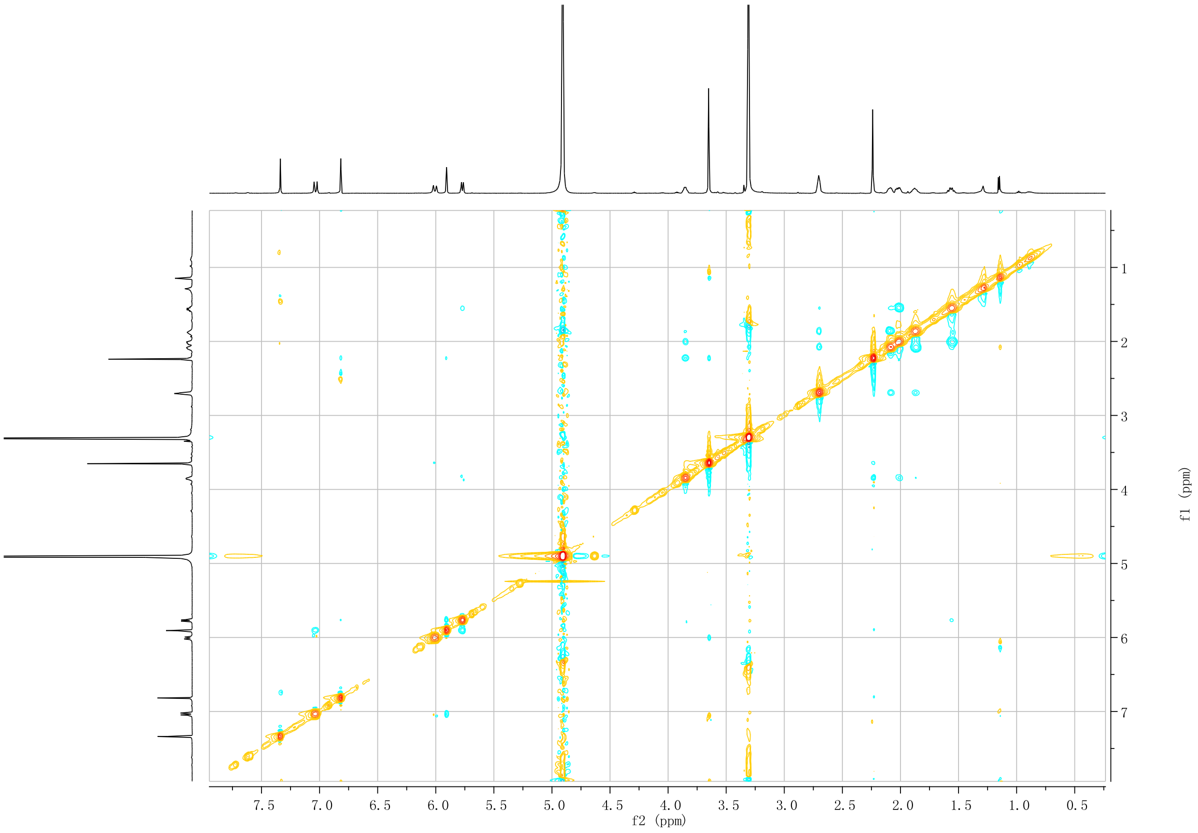

Figure S9: The NOE spectrum for compound **2**.

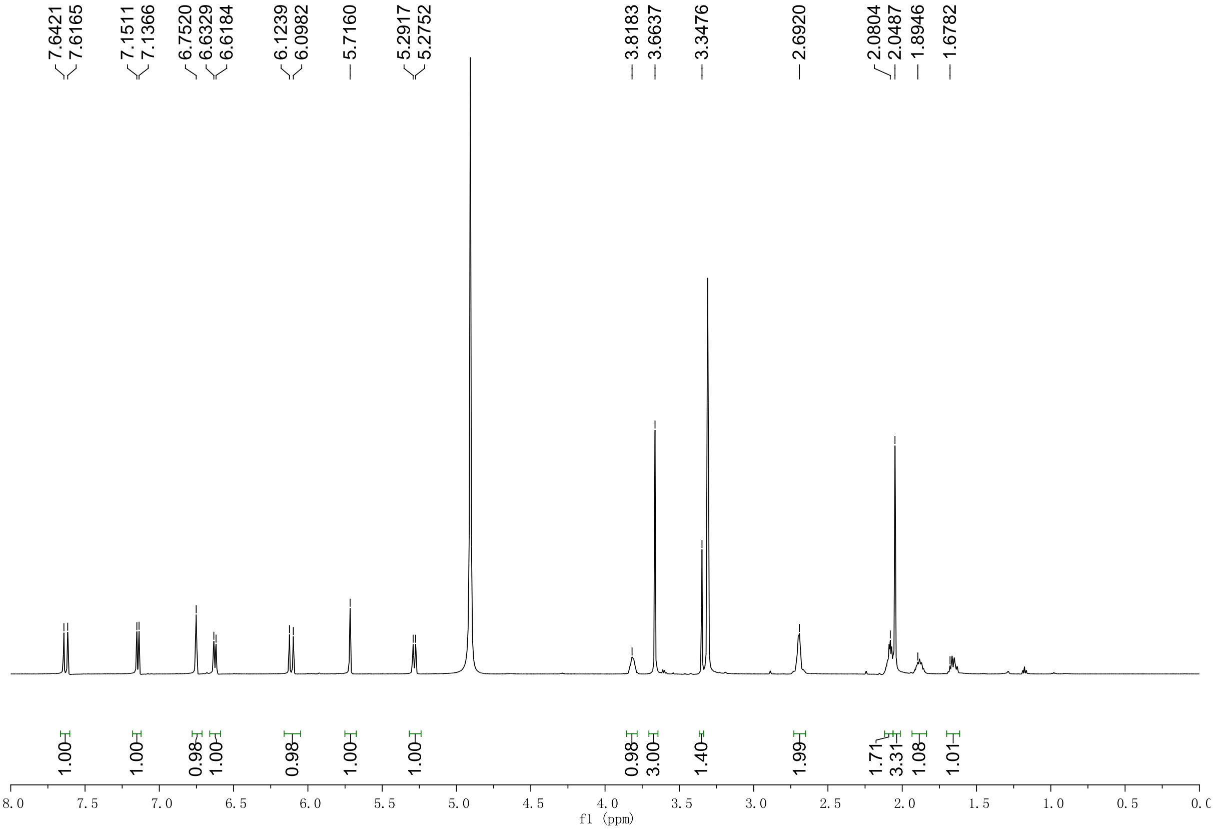

Figure S10: ^{1}H NMR (600 MHz, CD_3OD) spectrum for compound **3**.

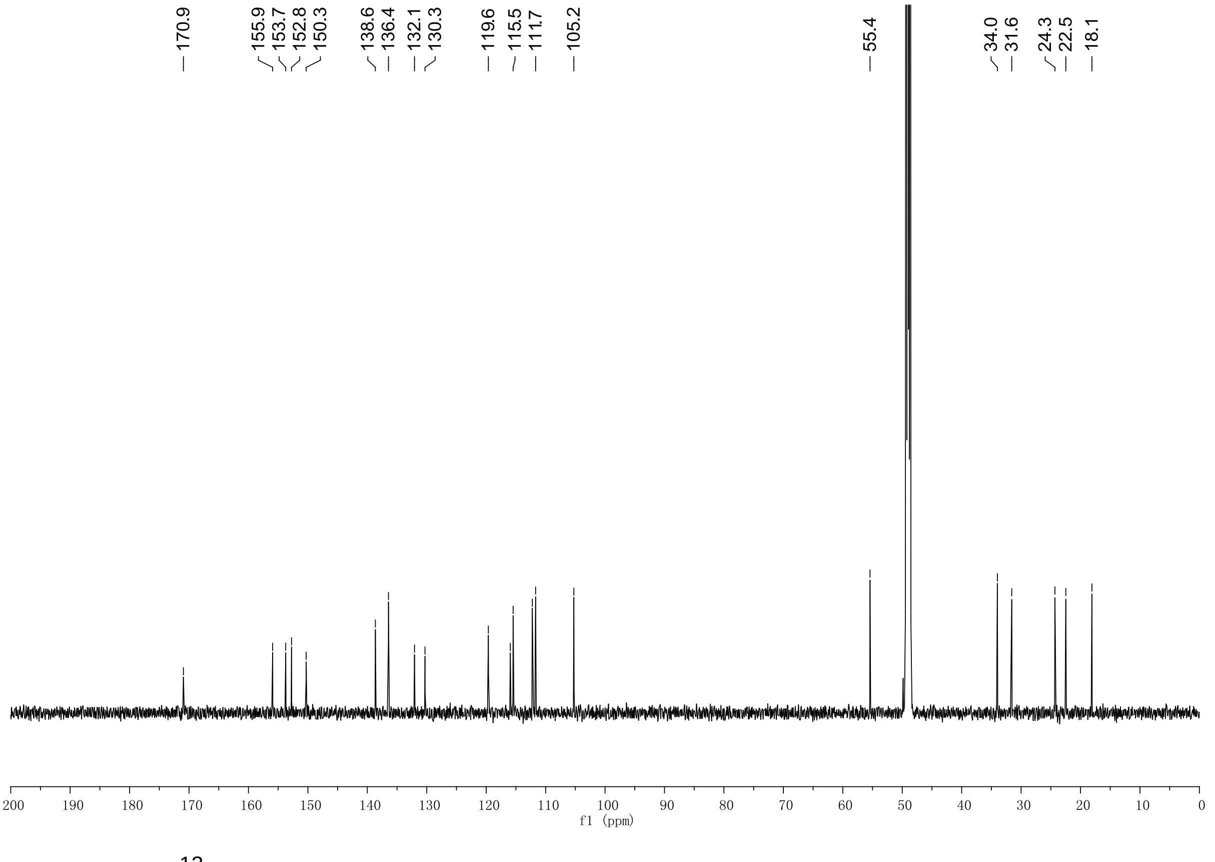

Figure S11. ^{13}C NMR (151 MHz, CD_3OD) spectrum for compound **3**.

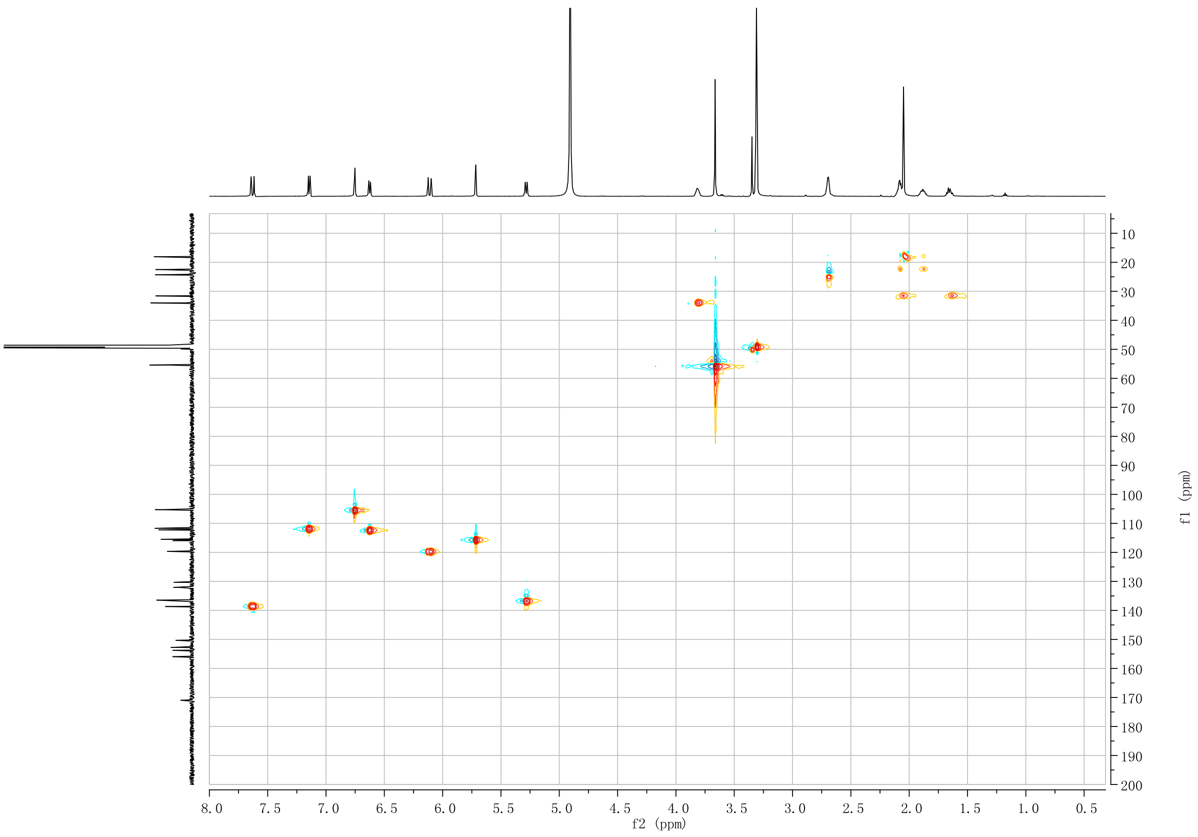

Figure S12: The HSQC spectrum for compound **3**.

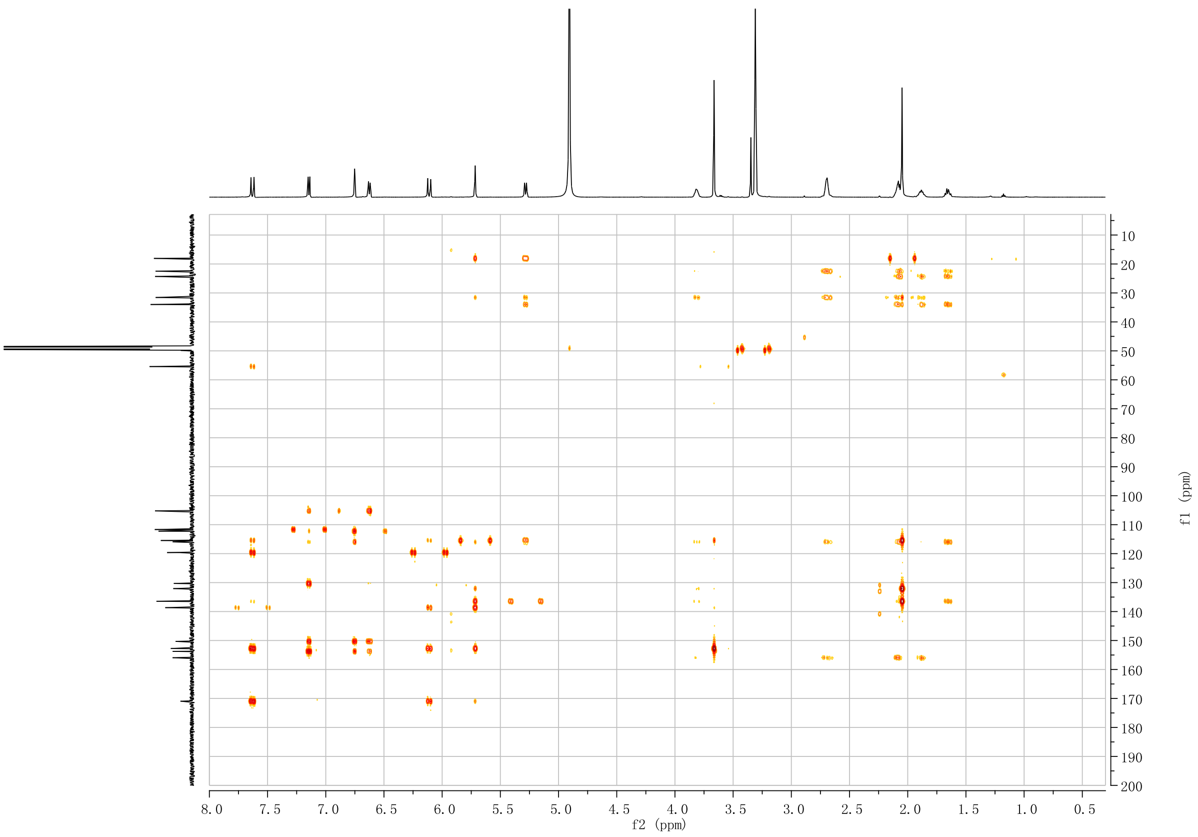

Figure S13: The HMBC spectrum for compound **3**.

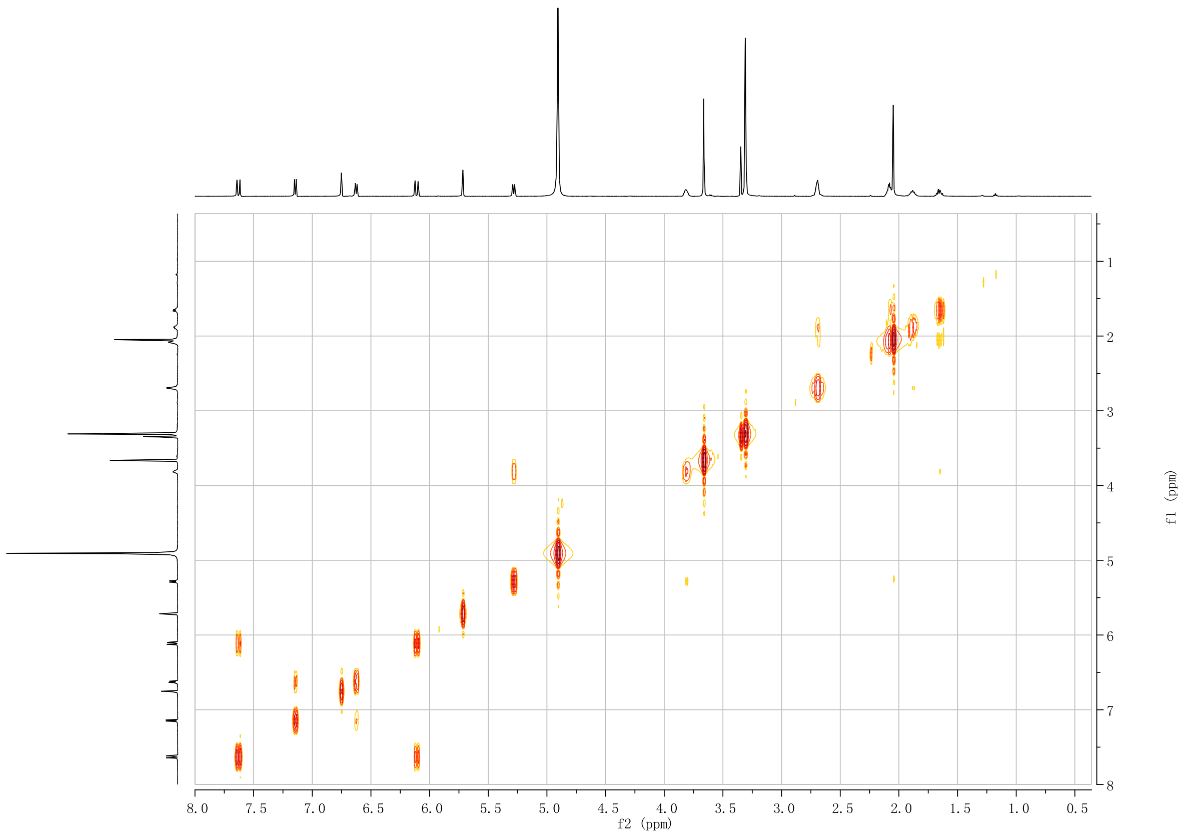

Figure S14: $^1H/^1H$ COSY spectrum for compound **3**.

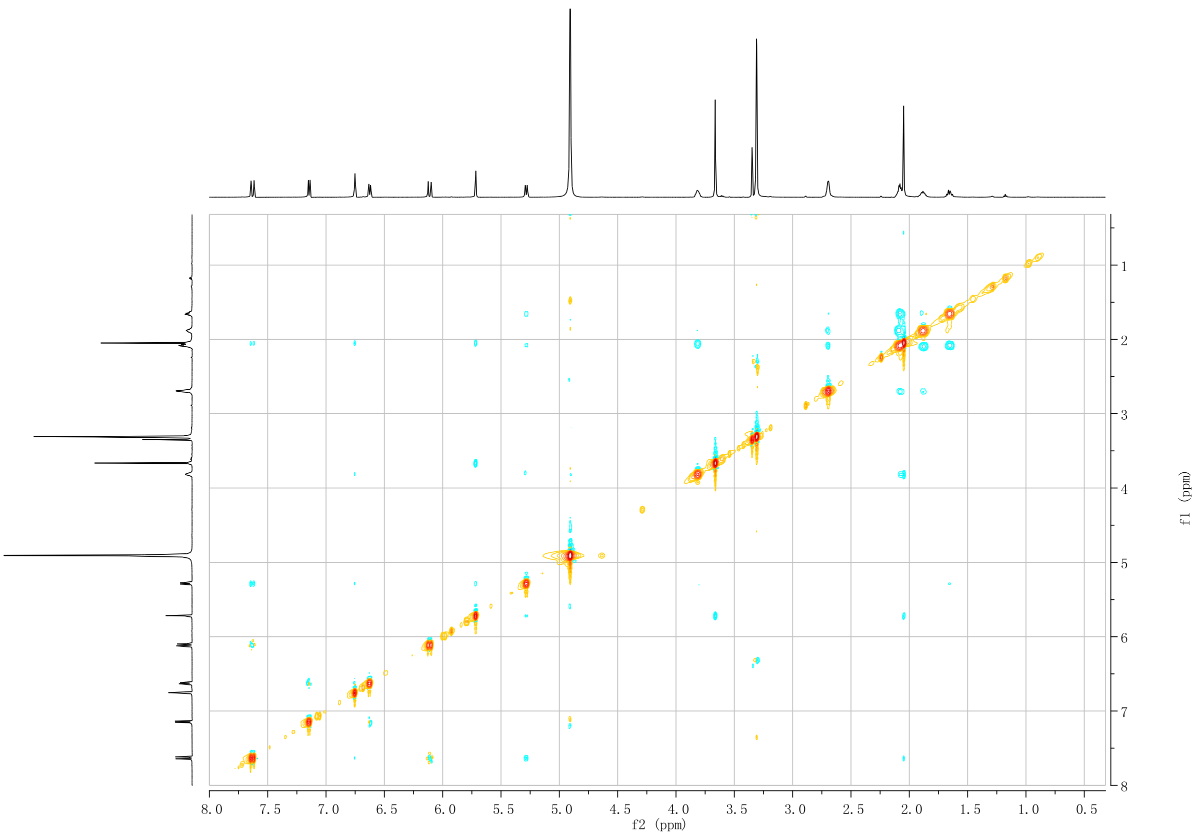

Figure S15: The NOE spectrum for compound **3**.

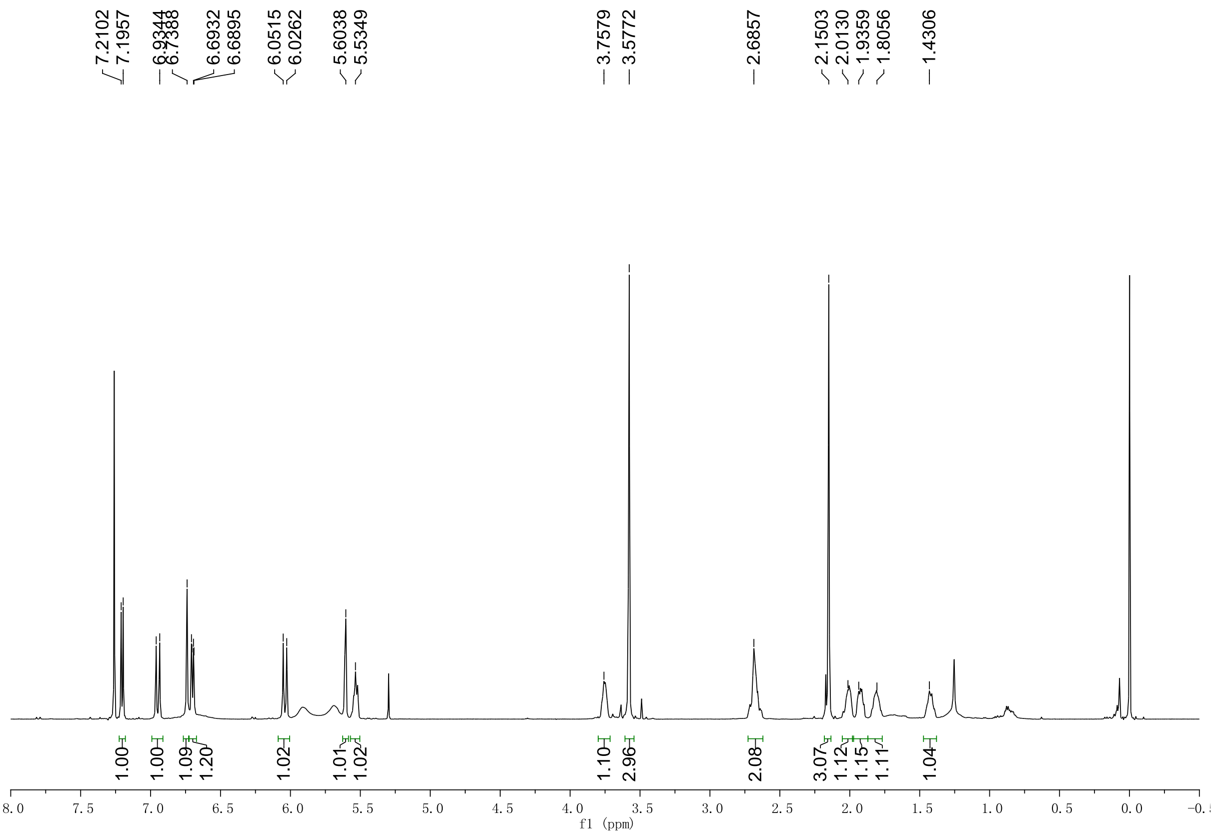

Figure S16: ^1^H NMR (600 MHz, $CDCl_3$) spectrum for compound **4**.

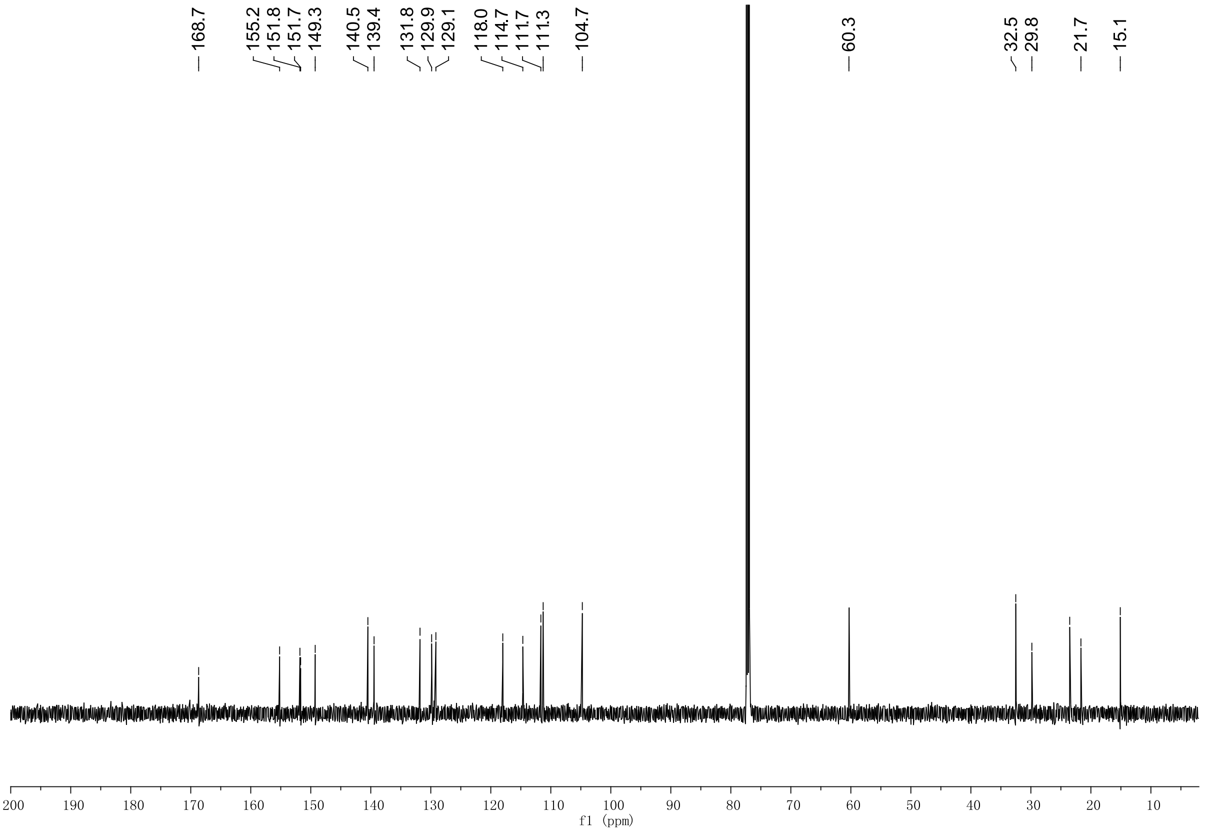

Figure S17: ^{13}C NMR (151 MHz, $CDCl_3$) spectrum for compound **4**.

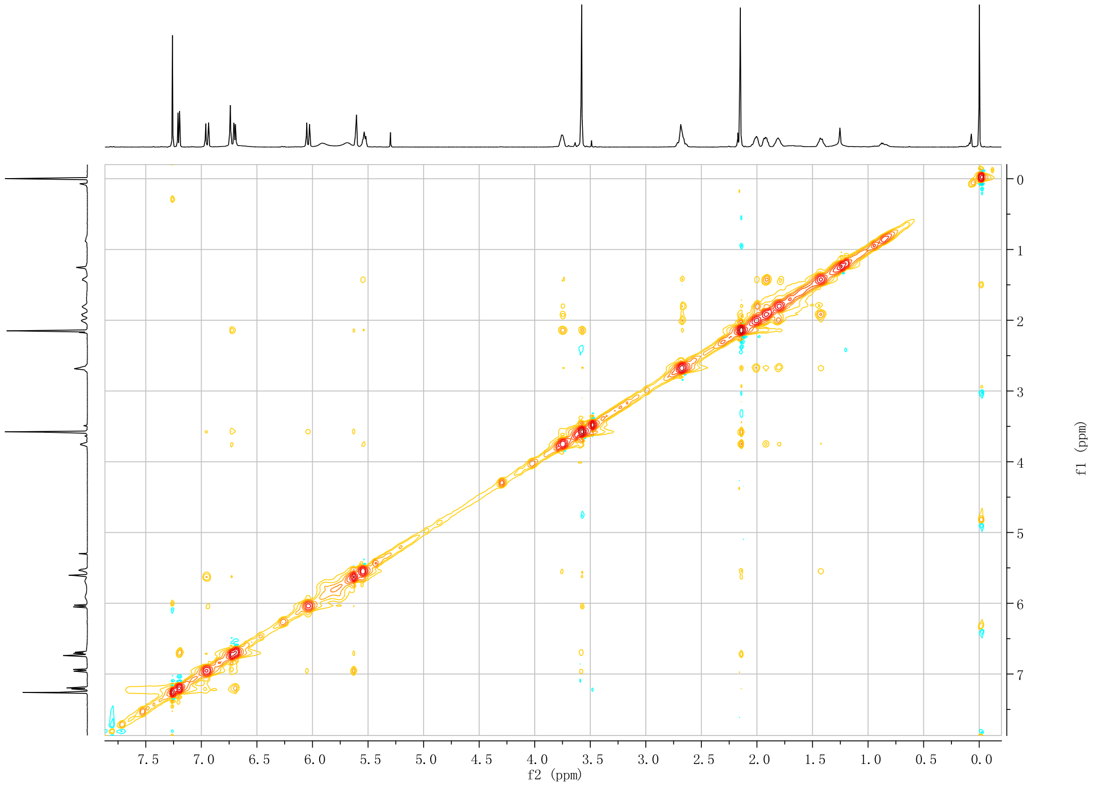

Figure S18: The NOE spectrum for compound **4**.

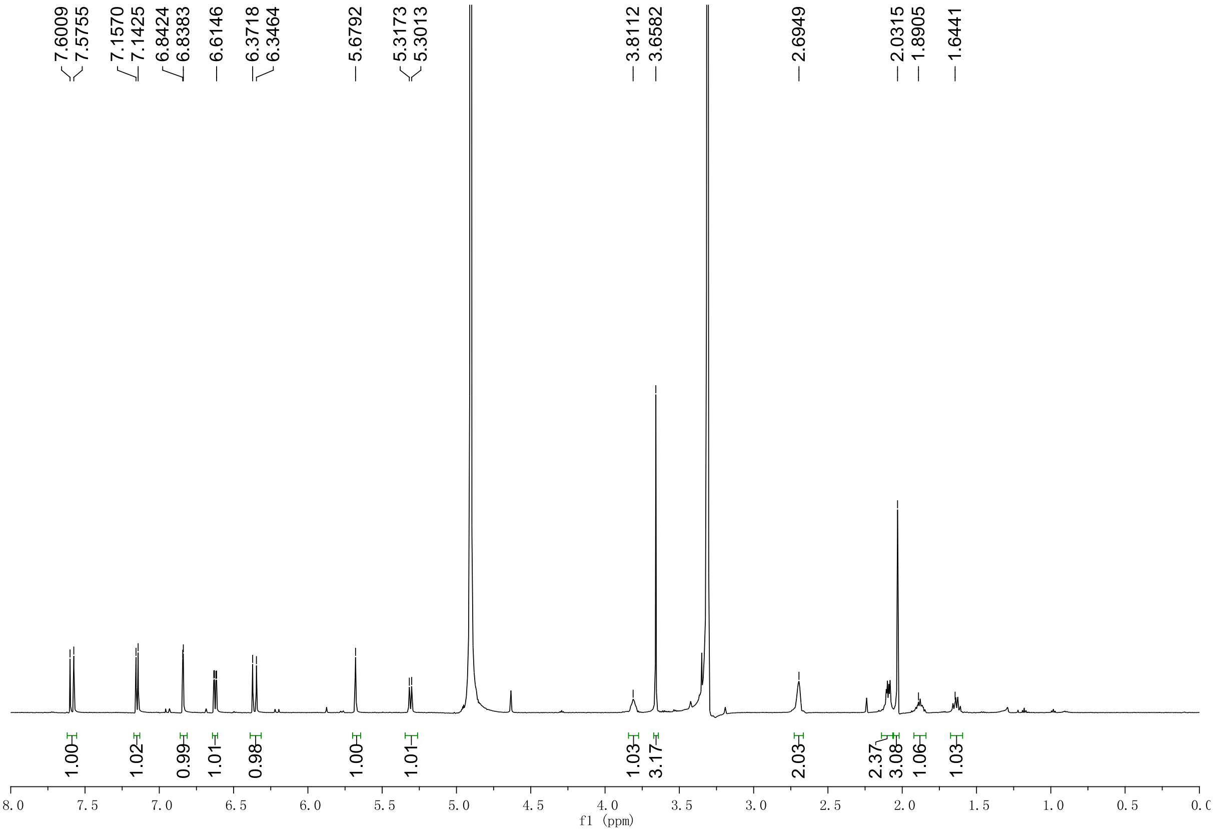

Figure S19: ^{1}H NMR (600 MHz, CD_3OD) spectrum for compound **5**.

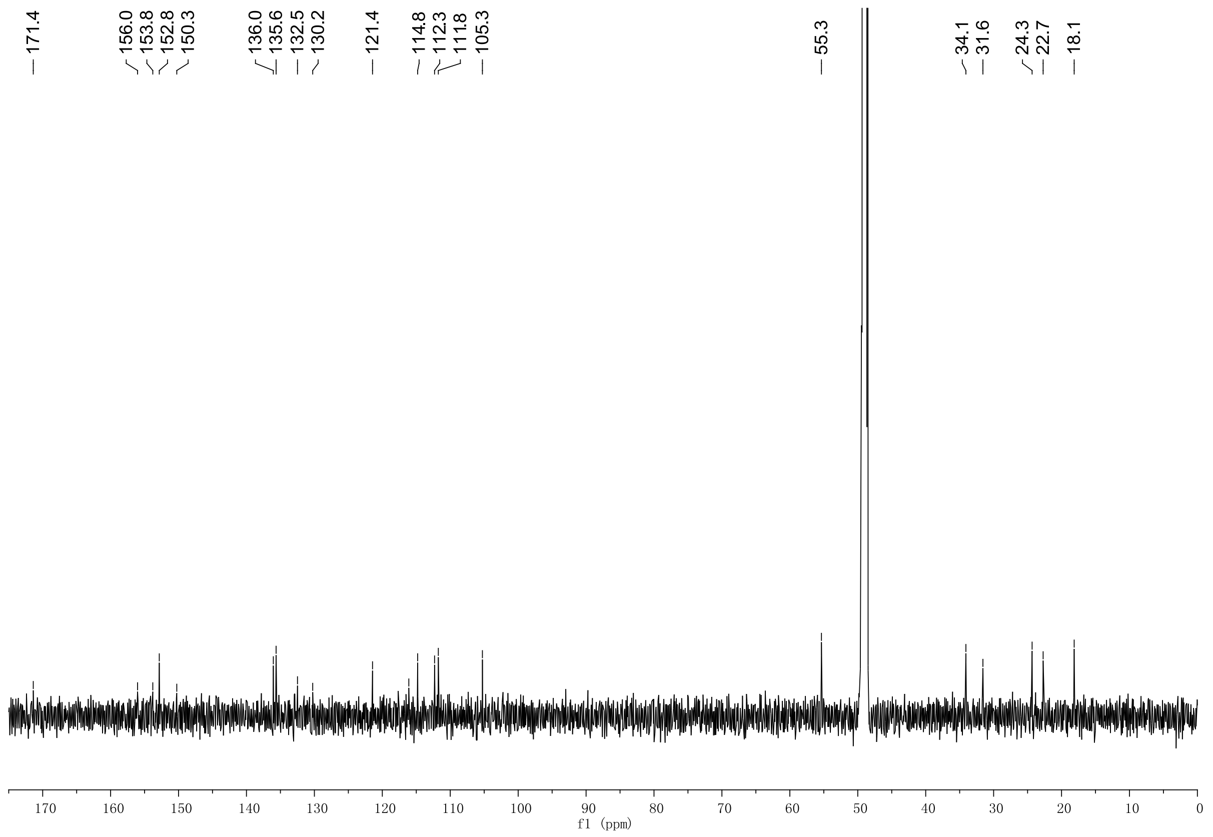

Figure S20: ^{13}C NMR (151 MHz, CD_3OD) spectrum for compound **5**.

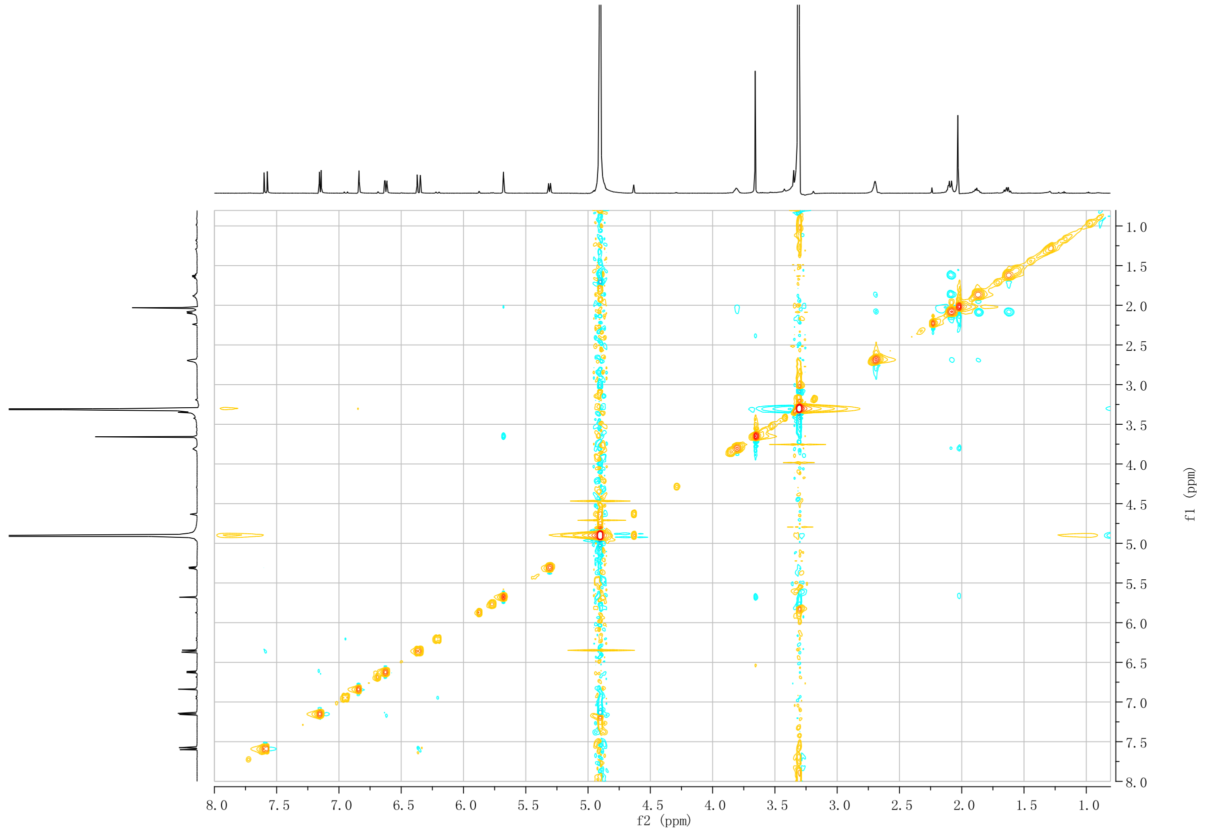

Figure S21: The NOE spectrum for compound **5**.

```
CLUSTAL W (1.83) multiple sequence alignment

KR5-ery      RAARTRWSPRGTVLVTGGTGGIGAHVARWLARSG-AEHLVLLGRRGADAPGASELREELT
KR6-ery      GTRESSWEPAGTALVTGGTGALGGHVARHLARCG-VEDLVLVSRRGVDAPGAAELEAELV
KR2-ery      TATSEYAVPGGTILVTGGTAGLGAEVARWLAGRG-AEHLALVSRRGPDTEGVGDLTAELT
KR1-ery      PATDDEWKPTGTVLVTGGTGGVGGQIARWLARRG-APHLLLVSRSGPDADGAGELVAELE
KR1-ave      THQPPTPTPHGTTLITGGTGALATHLTHHLTTHQPTQHLLLTSRTGPHTPHAQHLTTQLQ
KR5-ave      THQPPTPTPHGTTLITGGTGALATHLTHHLTTHQPTQHLLLTSRTGPHTPHAQHLTTQLQ
KR5-cuv      APTEPVAIGEGTVLITGGTGSLGSLVARHLVTEHGVRDLVLVSRQGPDADGATELTNELQ
                     ** *:****..:.  ::: *.     . .* * .* * .:  . .*  :*

KR5-ery      ALGTGVTIAACDVADRARLEAVLAAERAEGRTVSAVMHAAGVSTSTPLDDLTEAEFTEIA
KR6-ery      ALGAKTTITACDVADREQLSKLLEELRGQGRPVRTVVHTAGVPESRPLHEIGELES--VC
KR2-ery      RLGARVSVHACDVSSREPVRELVHGLIEQGDVVRGVVHAAGLPQQVAINDMDEAAFDEVV
KR1-ery      ALGARTTVAACDVTDRESVRELLGGIG-DDVPLSAVFHAAATLDDGTVDTLTGERIERAS
KR1-ave      QKGIHLTITTCDTSNPDQLQQLLNTIPPQH-PLTTVIHTAGILDDATLTNLTPTQLNNVL
KR5-ave      QKGIHLTITTCDTSNPDQLQQLLNTIPPQH-PLTTVIHTAGVNLFAPVSETDAESFSSVT
KR5-cuv      QHGARVRTLSCDLTDRTALAALIDETG----PLTGVVHTAGALADATVDHLDADALATTF
               *      :** :.   :  ::         :  *.*:*.     .:

KR5-ery      DVKVRGTVNLDELC-PDLDA--FVLFSSNAGVWGSPGLASYAAANAFLDGFARRRRSEGA
KR6-ery      AAKVTGARLLDELC-PDAET--FVLFSSGAGVWGSANLGAYSAANAYLDALAHRRRAEGR
KR2-ery      AAKAGGAVHLDELC-SDAEL--FLLFSSGAGVWGSARQGAYAAGNAFLDAFARHRRGRGL
KR1-ery      RAKVLGARNLHELT-RELDLTAFVLFSSFASAFGAPGLGGYAPGNAYLDGLAQQRRSDGL
KR1-ave      RAKAHSAHLLHQLT-QHTPLTAFVLYSSAAATFGAPGQANYAAANAYLDALAHHRHTHHL
KR5-ave      AAKATGAAILHELLLDHETLEHFILFSSGAGAWGSGNQCAYSAANAYLDALATHRQTHGL
KR5-cuv      APKANAAWWLHELT-QDQNLTLFMVFSSLAGVLGSAGQANYAAANSFLDALITHRRSHGL
               *. .:  *.:*   .     *:::** *.. *:     *:..*::**.:  :*:

KR5-ery      PVTSIAWGLWAGQ
KR6-ery      AATSVAWGAWAGE
KR2-ery      PATSVAWGLWAAG
KR1-ery      PATAVAWGTWAGS
KR1-ave      PATSIAWGTWQGN
KR5-ave      PGASIAWGPWAGK
KR5-cuv      PGTSLAWGSWERT
             . :::*** *
```

Figure S22: Alignment of modular PKS Ketoreductase (KR) domains. The Asp residue diagnostic of PKS KR specificity is labeled. Those containing the Asp are two known (KR1_ery and KR1_ave) to catalyze reduction with D configuration. Those without the Asp are known to catalyze ketoreduction with L configuration.
